# Prenatal testosterone-induced fetal growth restriction is associated with down-regulation of rat placental amino acid transport

**DOI:** 10.1186/1477-7827-9-110

**Published:** 2011-08-03

**Authors:** Kunju Sathishkumar, Rebekah Elkins, Vijayakumar Chinnathambi, Haijun Gao, Gary DV Hankins, Chandra Yallampalli

**Affiliations:** 1 Department of Obstetrics and Gynecology, The University of Texas Medical Branch Galveston, Texas, USA

## Abstract

**Background:**

Exposure of pregnant mothers to elevated concentrations of circulating testosterone levels is associated with fetal growth restriction and delivery of small-for-gestational-age babies. We examined whether maternal testosterone crosses the placenta to directly suppress fetal growth or if it modifies placental function to reduce the capacity for transport of nutrients to the fetus.

**Methods:**

Pregnant rats were exposed to testosterone propionate (TP; 0.5 mg/kg) by daily subcutaneous injection from gestational days (GD) 15-19. Maternal and fetal testosterone levels, placental nutrient transport activity and expression of transporters and birth weight of pups and their anogenital distances were determined.

**Results:**

This dose of TP doubled maternal testosterone levels but had no effect on fetal testosterone levels. Maternal daily weight gain was significantly lower only on GD 19 in TP treated dams compared to controls. Placental weight and birth weight of pups were significantly reduced, but the anogenital distance of pups were unaffected by TP treatment. Maternal plasma amino acids concentrations were altered following testosterone exposure, with decreases in glutamine, glycine, tyrosine, serine, proline, and hydroxyproline and increases in asparagine, isoleucine, leucine, lysine, histidine and arginine. In the TP dams, placental system A amino acid transport activity was significantly reduced while placental glucose transport capacity was unaffected. Decreased expression of mRNA and protein levels of *slc38a2*/Snat2, an amino acid transporter, suggests that reduced transporter proteins may be responsible for the decrease in amino acid transport activity.

**Conclusions:**

Taken together, these data suggest that increased maternal testosterone concentrations do not cross the placenta to directly suppress fetal growth but affects amino acid nutrient delivery to the fetus by downregulating specific amino acid transporter activity.

## Background

Elevated testosterone levels during pregnancy is shown to be associated with low birth weight in humans and animals [1-5]. Women experiencing hyperandrogenism associated with polycystic ovarian syndrome (PCOS) [6,7] and preeclampsia [8-10] have a higher-than-normal prevalence of small-for-gestational age deliveries [11-15]. In animal models, such as rat and sheep, testosterone exposure during pregnancy leads to a dose-dependent reduction in birth weight of fetuses [1,3,16,17]. In addition to causing low birth weight, hyperandrogenemia during pregnancy also poses an increased risk for the development of cardiovascular and metabolic dysfunctions later in life for both the child and the mother [18-21]. Despite findings that excess androgen during pregnancy influences fetal growth restriction and programming of metabolic tissues, there are no studies that have examined the underlying mechanisms. An understanding of such mechanisms will aid in the development of effective interventions to improve mother's health and decrease or perhaps prevent conditions that lead to the birth of small-for-gestational-age babies and the consequent risk of complications in adult life.

Testosterone exposure during pregnancy in rats and sheep does not affect the mother's food intake [17,22] or alter levels of other important pregnancy-related hormones, such as estradiol, progesterone, corticosterone, insulin, leptin, thyroid hormones (total T3, total T4, and free T4) and IGF1 [17,22]. Testosterone exposure during pregnancy in rats and sheep was also found not to affect maternal blood levels of glucose, triglycerides and cholesterol [22,23]; thus, testosterone-induced fetal growth restriction may not be secondary to alterations in the mother's metabolic status.

Testosterone is lipophilic and is suggested to diffuse through the placenta [24-26] to exert a direct effect on fetal growth and/or energy homeostasis. Alternatively testosterone may affect normal placental development and function [27]. The crucial role of the placenta during fetal development is thought to depend on the transplacental exchanges of oxygen and nutrients, as well as waste between the closely apposed maternal and fetal circulatory systems [28]. The placenta is also an endocrine target, expressing a broad spectrum of hormone receptors including androgen receptors [29]; hence, testosterone may modify placental function and reduce the capacity for transport of nutrients to the fetus. In this study, we evaluated whether maternal testosterone crosses the placenta to directly affect fetal growth or testosterone modifies placental development and function to affect nutrient transport capacity to the fetus. Our studies show that an increase in circulating maternal testosterone levels in pregnant rats at a clinically relevant concentration (2-fold--similar to that observed in human pregnancies complicated with IUGR [6-10]) induced fetal growth restriction without increase in fetal testosterone levels, but it is associated with a reduction in placental amino acid transport activity, possibly through a decrease in expression of the *slc38a2*/Snat2 amino acid transporter.

## Methods

### Animals

Timed-pregnant Sprague-Dawley rats (Harlan, Houston, TX) were received on gestational day (GD) 12 (GD 1 = day of sperm-positive smear) and housed 2 per cage in polycarbonate cages. They were acclimated to 68-74°F and 40-50% relative humidity (12 hours, light:12 hours, dark) and given a Prolab RMH 2500 diet (high-energy diet for gestation and lactation) and tap water *ad libitum*. On GD 14, the dams were randomly assigned to 2 treatment groups. The pregnant rats received daily doses from GD 15-19 by subcutaneous (sc) injection with either vehicle (n = 24) or 0.5 mg/kg TP (n = 24; Sigma, St. Louis, MO) suspended in 0.1 ml sesame oil. We and others have shown that TP treatment to pregnant rats leads to a dose-dependent reduction in birth weight of fetuses [1,17,30]. For this study, we elected to use this dosing regimen, since it caused a 2-fold increase in circulating maternal testosterone levels [17] similar to that observed in human pregnancies complicated with fetal growth inhibition [6-10]. Because an increase in testosterone levels during the later stage of pregnancy was shown to be associated with IUGR [5] and preeclampsia [31,32], we chose this time period (GD 15-19) of TP treatment in pregnant rats.

Maternal weight was monitored throughout the dosing period. In *Experiment #1*, the pregnant rats were sacrificed 2 hours after last TP dose on GD 19 (at plateau circulating testosterone concentration [1,33,34] to measure maternal/fetal testosterone levels; n = 6 in each group). In *Experiment #2*, the pregnant rats were sacrificed on GD 21 (the period of active placental function) to determine placental nutrient transport capacity (n = 6, each group). In *Experiment #3*, spontaneous vaginal delivery was allowed for some dams (n = 6, each group) to determine birth weight and anogenital distance (AGD), an indicator of androgenicity [35]. All procedures were approved by the Institutional Animal Care and Use Committee at The University of Texas Medical Branch (UTMB).

### Experimental Design

#### Experiment 1: Maternal/fetal testosterone Levels

On GD 19, 2 hours after dosing, 6 control and 6 TP dams were euthanized by CO_2 _asphyxiation. Maternal blood and fetuses were collected no earlier than 2 hours after dosing to allow the TP to undergo distribution and metabolism and reach plateau testosterone levels in both the dams and fetuses [1,33,34]. Each dam was euthanized and its fetuses collected before the next dam was euthanized. Maternal blood was collected by heart puncture. Fetuses were removed from the uterus, held on ice in a small plastic petri dish, and saved in 15-ml plastic, round-bottomed Falcon tubes (Becton-Dickinson, Lincoln Park, NJ). Fetuses were stored at -20°C for ~1 week at which time they were extracted and assayed for testosterone levels. Maternal blood was centrifuged and plasma was stored at -70°C for 1 week until it was assayed for testosterone levels. Storage of fetuses or plasma for this time period did not cause any significant loss in testosterone levels. In addition to the above mentioned TP dose (0.5 mg/kg/day; n = 5), a lower dose (0.1 mg/kg/day; n = 5) and a higher dose (2.5 mg/kg/day; n = 5) were also administered to a separate set of dams where no change or a definitive increase in maternal/fetal testosterone level, respectively was expected.

##### DNA extraction from fetus and sex determination

Genomic DNA was extracted from a piece of fetal tissue (hind limb) and tails of adult male and female rats (as controls) with Qiagen DNeasy Blood & Tissue Kit (Qiagen, Inc., Valencia, CA) according to manufacturer's protocol. Sex determination was described by Kuddus et al [36]. Males were determined by the presence of the *Sry *gene in genomic DNA with 1 μg DNA template added in polymerase chain reactions (PCR) and females by no *Sry *gene amplification. The sequence of forward primers for the *Sry *gene was 5'-cacaagttggctcaacagaatc-3' and reverse primer 5'-agctctactccagtcttgtccg-3'. Genomic DNA (1 μg) from adult males and females was included as either a positive or negative control for the PCR procedure. The PCR conditions were as follows: 1) 94°C for 5 minutes; 2) 94°C for 1 minute, 54°C for 2.5 minutes, and 72°C for 1 minute for 36 cycles; and 3) 72°C for 7 minutes.

##### Fetal testosterone extraction

Testosterone was extracted from the fetus as described previously [1,37]. Fetuses were thawed and homogenized individually in 500-μl distilled and deionized water with a Polytron homogenizer (Brinkmann Instruments, Westbury, NY). After homogenization, 3 ml ethyl ether (Fisher Scientific, Pittsburgh, PA) was added to each tube, and tubes were vortexed for 30 seconds, and centrifuged at 2000 rpm (1000 × g) at 8°C for 10 minutes. Following centrifugation, each tube was held 1 at a time in an acetone/dry ice bath until the bottom aqueous layer froze, and the supernatant (ether layer) was then transferred to a 12- × 75-mm glass tube. Ether extraction was performed twice, and glass tubes of ether extract were dried in a fume hood overnight. Tubes were stored at -20°C until analysis by radioimmunoassay.

##### Radioimmunoassay (RIA) of testosterone

Each tube of dried fetal extract was re-suspended by vortexing for 30 seconds in 70 μl of assay buffer provided in the Testosterone double antibody RIA kit #07189102 (MP Biomedicals, Solon, OH). Next, 50 μl of the 70-μl fetal resuspension were transferred to the tubes in the RIA kit, and testosterone levels were determined according to the manufacturer's protocol. Tubes were read for 1 minute each in a Cobra gamma counter (Packard Instrument Co., Downers Grove, IL). The maternal serum was vortexed, and 50 μl of undiluted and diluted plasma (1:2) assayed in duplicate by RIA for testosterone following the testosterone RIA kit protocol. Fetal testosterone levels were expressed as ng/g of fetus (these data represent 3 males or females per litter per dose group) and maternal plasma testosterone levels were expressed as ng/ml. Intra- and interassay coefficients of variation for testosterone were 3% and 5% and sensitivity was 0.04 ng ml^-1^.

#### Experiment 2: Placental nutrient transport capacity

On GD 21, maternal blood and placenta were collected from control and TP dams (n = 6 in each group). Maternal plasma was used for determination of amino acid concentrations and placentas were processed for protein and RNA analysis. The placental RNA and protein samples of each dam constituted a pooled sample isolated from 3 placentas per dam. In a separate set of control and TP pregnant rats, placental transport measurements were carried out (n = 6 in each group).

##### Analysis of amino acid concentrations in maternal plasma

Plasma amino acids were measured at Protein Chemistry core lab at UTMB. Briefly, free amino acids were analyzed by using ion exchange HPLC (Hitachi model L-8800 amino acid analyzer; Hitachi, Inc., Pleasanton, CA) equipped with columns (number 2622: 4.6 × 40 mm + 4.6 × 40 mm, Hitachi, Tokyo, Japan) and by gradient elution with MCI buffer L-8500-PF kit (Wako, Osaka, Japan). Amino acids post labeled with ninhydrin were detected by measuring absorbance at 440 and 570 nm.

##### Implantation of vascular catheters

Transport measurements were carried out as previously described for the awake pregnant rats [38]. Animals were anesthetized with a mixture of ketamine (45 mg/kg; Burns Veterinary Supply, Westbury, NY) and xylazine (5 mg/kg; Burns Veterinary Supply) intraperitoneally. A 2-cm midline incision was performed, and the right carotid artery and right jugular vein were localized and catheterized with PE 50 tubing. Subsequently, catheters were tunneled subcutaneously to the neck where they emerged, filled with heparinized saline (500 IU ml^-1^), and plugged. After awakening, animals were returned to the animal facilities where they were left overnight to fully recover.

##### Transport measurements

Placental transport was studied as described by Coan et al [38], using the intravenous administration of 3-*O*-methyl-D-[^3^H]glucose (50 μCi kg^-1^) and [^14^C]methylaminoisobutyric acid (10 μCi kg^-1^). 3- *O *-Methyl-D-glucose and α-(methylamino)isobutyric acid are nonmetabolizable model substrates used for characterization of the D-glucose and system A amino acid transport system, respectively. These isotopes are extensively used to examine glucose and amino acid transport across placenta [38,39]. After the administration of isotopes, the rat was placed in a cage where it was free to walk around for 6 minutes, when an arterial blood sample (0.5 ml) was withdrawn for determination of radioactivity in plasma. Seven minutes after the infusion of isotopes, 1 ml ketamine (50 mg ml^-1^) was injected into the venous catheter to kill the animal. This time point was chosen based on previous reports [38] demonstrating low feto-maternal backflux of isotopes up to at least 7 minutes after isotope injection. The placentas and fetuses were weighed individually. Subsequently, fetuses and placentas from each litter were pooled and cut into small pieces and homogenized in three volumes of distilled water. Homogenates were mixed with trichloroacetic (20%, 1:3), and the vials were centrifuged at 12 000 g for 10 minutes. Liquid scintillation fluid (12 ml; Aquasafe 300 plus, Zinsser Analytic, Frankfurt, Germany) was added to 3 ml of supernatant. Distilled water (3 ml) was added to 150-μl plasma samples, followed by 12 ml of scintillation fluid. Vials were shaken for 30 minutes prior to β-counting in a liquid scintillation counter.

##### Real-time PCR

Total RNA was extracted from placenta by using RNeasy extraction kits (Qiagen, Valencia, CA). The RNA (1 μg) was reverse transcribed into cDNA (QuantiTect Rev. Transcription Kit; Qiagen). Quantitative real-time PCR analysis was performed with an ABI-PRISM 7700 real-time machine (Applied Biosystems Inc., Foster City, CA), using a SYBR Green detection system (Applied Biosystems) at UTMB's Sealy Center for Cancer Cell Biology core facility. Published primer sequences [40] were used to measure the relative mRNA amounts of the *slc38a1, 2*, and *4*. The reactions were first incubated at 50°C for 30 min followed by 95°C for 15 min and then amplification of 35 cycles of each at 95°C for 15 s, 60°C for 60 s for *slc38a1*, and *2*, and 62.8°C for 60 s for *slc38a4*. The primers utilized were *slc38a1*: (forward 5'-TCAGCCTGGTACGTCGATGG-3', reverse 5'-CCAGGTTCTTCAAGAGACACAG-3'), *slc38a2*: (forward 5'-AGAGCAATTCCAGTATTAGC-3', reverse 5'-TTAATCTGAGCAATGCGATTGTG-3'), and *slc38a4 *(forward 5'-GGCAGTGGTGTGGAGTACGAAGC-3', reverse 5'-TGGAATCGCGTAGGCCGTG-3'). After PCR, melting curves were acquired by stepwise increase of the temperature from 55°C to 95°C to ensure that a single product was amplified in the reaction.

##### Western blot

Placentas were homogenized on ice in buffer containing (mM): Tris-Hepes 10, sucrose 250, and EDTA 1, and 1.6 μM antipain, 0.7 μM pepstatin, and 0.5 μg ml^-1 ^aprotinin. Protein concentrations were determined by the Bradford assay. Equal amounts of protein (25 μg) were loaded onto 10% polyacrylamide gels and electrophoresed at 200 V. The separated proteins were transferred overnight onto a nitrocellulose membrane. The membranes were blocked in blocking buffer (5% nonfat dry milk) and then incubated overnight with primary antibodies (1:500 for Snat1, 1:5000 for Snat2 and 1:1000 for Snat4; Snat1 and Snat4 antibody was a generous gift from Drs. Jean Jiang and Thomas Jansson, respectively, University of Texas Health Science Center San Antonio. Snat2 antibody was a generous gift from Dr. P.D. Prasad, Medical College of Georgia). Peroxidase-labeled, anti-rabbit IgG (1:1000; Vector Laboratories, Burlingame, CA) was used as a secondary antibody. The final detection was accomplished by using enhanced chemiluminiscence (Pierce Biotechnology, Rockford, IL) to visualize signals on autoradiographic film (Hybond, Amersham). The relative density of the bands was evaluated by densitometry with alpha-ease software. All chemicals were purchased from Sigma-Aldrich Co. (St. Louis, MO) unless otherwise noted.

#### Experiment 3: Birth weight of pups and their anogenital distances

Pregnant rats were allowed to deliver spontaneously. Immediately after birth, the pups (n = 6 litters in each group) were counted, weighed, and sexed based on anogenital distances (AGD). The AGD in each male and female pup was measured in a blind fashion under a dissecting microscope using calipers [41].

### Statistical analysis

Transport data were presented as placental disintegrations per minute (dpm) per gram placenta (representing placental uptake of isotope), fetal dpm per gram fetus (representing the amount of isotope transported per gram fetus), and fetal dpm per gram placenta (giving a measure of the amount of isotope transported per gram placenta, i.e., the relative transport capacity of the placenta). Transport data were expressed for the TP group in relation to the controls where control values were arbitrarily assigned a value of 1. To identify appropriate endogenous housekeeping gene for analysis of real time PCR data, the mRNA expression of several housekeeping genes (beta actin, beta-2-microglobulin, glyceraldehyde-3-phosphate dehydrogenase, succinate dehydrogenase complex (SDHA), ubiquitin C, and 18s rRNA) were performed. In our experimental conditions, 18s and SDHA were found to be stably expressed across control and TP placentas. Hence the abundance of the target sequences was calculated relative to 18S rRNA using the following formula: relative abundance = 2^-ΔCT^, where ΔC_T _is calculated as the difference between the C_T _(threshold cycle) of the test sequences and of the reference 18S rRNA sequence.

Since observations in individual pups and placentas of the same litter were not independent, an average was obtained for each litter. Therefore, n = 1 represents averaged values in one litter, and all data presented as mean ± S.E.M. Two-way ANOVA followed by Bonferroni post hoc test was performed on multiple observations and unpaired Student's *t*-test for comparison of single observations between control and TP groups. Data analysis was done using GraphPad Prism for Windows (GraphPad Software, San Diego, CA). Differences were considered statistically significant at *P *< 0.05.

## Results

**Figure 1:**
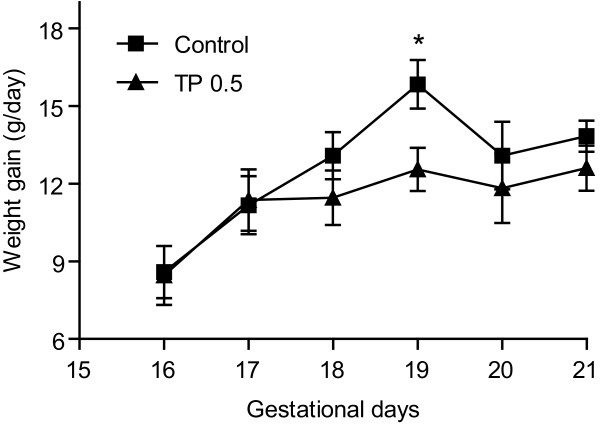
**Daily weight gain in control and TP dams**. TP was administered prenatally (0.5 mg/kg; gestational days (GD) 15-19). Weight of dams was measured and expressed as g/day (n = 10, Control; and n = 11, TP). Data were expressed as mean ± SEM. *P < 0.05 vs controls.

Maternal total weight gain during the dosing period was not significantly different (*P *= 0.25) between control (61.4 ± 3.54; n = 10) and TP dams (56.8 ± 2.03; n = 13). However, when expressed as daily weight gain through the dosing period, TP dams gained significantly less (*P *< 0.01) weight on GD 19 (12.5 ± 0.84 grams; n = 11) compared to controls (15.8 ± 0.93 grams; n = 10) (Figure 1). There was no significant difference in litter size between control (12.1 ± 0.46) and TP (11.3 ± 0.52) groups.

### Experiment 1: Maternal and fetal testosterone levels

**Figure 2:**
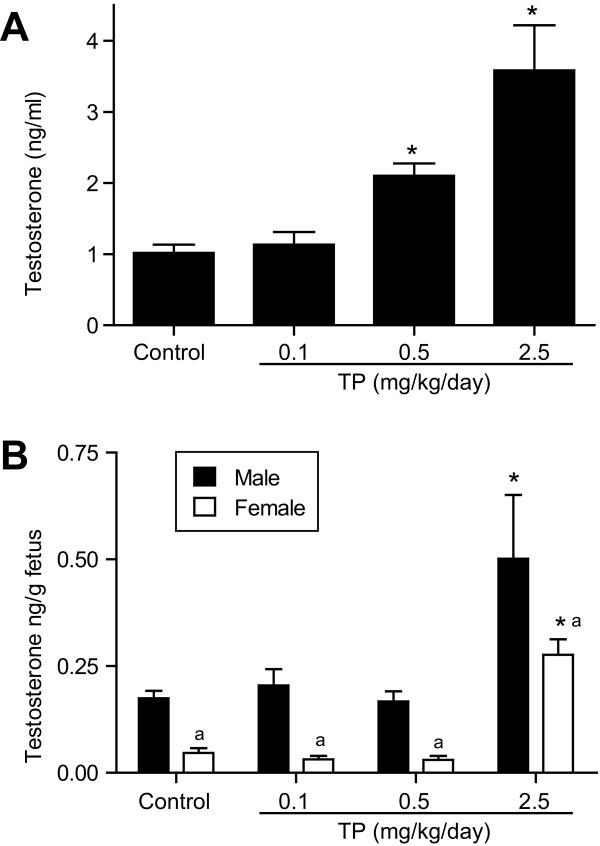
**Maternal and fetal testosterone levels in control and TP groups**. Testosterone levels in dams and fetuses at gestational day (GD) 19, after subcutaneous administration of 0, 0.1, 0.5, or 2.5 mg TP/kg (GD 15-19; n = 5-6). (**A**) Testosterone levels in dam plasma. (**B**) Testosterone levels in fetal homogenates. Values represent litter means. Data are expressed as mean ± SEM. *P < 0.05 vs controls.

Maternal serum testosterone levels (in ng/ml) increased in a dose-dependent fashion and were significantly elevated (*P *< 0.05) at 0.5 (n = 6) and 2.5 mg/kg TP (n = 5) (Figure 2A). In contrast, fetal testosterone levels (in ng/fetus) rose significantly (*P *< 0.05) only in the males and females at 2.5 mg TP (n = 5) and not at 0.5 mg TP (n = 6) (Figure 2B), despite the increased testosterone levels in the dam at this dose. The testosterone level in the male fetuses was higher than that in the females across all treatments, a finding consistent with previous reports [42].

### Experiment 2: Placental nutrient transport function

#### Maternal plasma amino acid concentrations

There were no significant differences in the plasma concentration of some amino acids between control and TP dams. Asparagine (+61%), isoleucine (+30%), leucine (+27%), lysine (+37%), histidine (+63%) and arginine (+75%) were significantly increased (*P *< 0.05) in TP dams (n = 6) compared to controls (n = 6) (Table 1). On the other hand, glutamine (-16%), glycine (-31%), serine (-24%), tyrosine (-12%), proline (-23%), and hydroxyproline (-80%) were significantly reduced (*P *< 0.05) in TP-exposed dams (n = 6) compared with controls (n = 6) (Table 1).

**Table 1:** Amino acid concentrations in maternal plasma of control (n = 6) and testosterone-exposed dams (n = 6).

Amino acid	Control (n = 6)	TP (n = 6)	*p *value
Taurine	0.13 ± 0.001	0.13 ± 0.005	1
Phosphoserine	1.58 ± 0.059	1.30 ± 0.211	0.23
Aspartic acid	0.25 ± 0.037	0.25 ± 0.008	1
Therionine	3.66 ± 0.076	3.62 ± 0.167	0.83
Asparagine	0.72 ± 0.070	1.16 ± 0.080*	0.002
Glutamic acid	2.24 ± 0.423	2.65 ± 0.295	0.45
Glutamine	12.86 ± 0.227	10.82 ± 0.581*	0.008
Glycine	3.07 ± 0.142	2.11 ± 0.138*	0.0007
Alanine	7.48 ± 0.186	7.14 ± 0.230	0.28
Citrulline	0.90 ± 0.174	0.88 ± 0.087	0.92
Valine	1.67 ± 0.161	2.08 ± 0.219	0.16
Methionine	0.57 ± 0.026	1.10 ± 0.253	0.06
Cystine	0.14 ± 0.015	0.27 ± 0.073	0.11
Isolucine	0.93 ± 0.051	1.21 ± 0.088*	0.02
Leucine	1.61 ± 0.048	2.05 ± 0.174*	0.03
Tyrosine	0.66 ± 0.022	0.74 ± 0.015*	0.01
Phenylalanine	0.90 ± 0.092	0.97 ± 0.065	0.55
Ornithine	0.73 ± 0.009	0.86 ± 0.135	0.36
Lysine	7.68 ± 1.19	10.58 ± 0.431*	0.04
Histidine	0.30 ± 0.023	0.49 ± 0.059*	0.01
Arginine	1.61 ± 0.235	2.81 ± 0.035*	0.0005
Serine	4.05 ± 0.138	3.09 ± 0.139*	0.0006
Hydroxyproline	0.55 ± 0.034	0.114 ± 0.043*	≤ 0.0001
Proline	3.54 ± 0.229	2.72 ± 0.162*	0.015

*P < 0.05 *versus *control.

#### Fetal and placental weights

On GD 21, the fetal weights were significantly lower by 7% and placental weights were significantly lower by 11% (*P *< 0.05; n = 6 in each group) in the TP group compared with controls (Table 2). There was no significant difference in fetal-to-placental weight ratio between control and TP groups (Table 2).

**Table 2:** Litter size, and fetal and placental weights on GD21.

Group	*n* litters	litter size (*n*)	fetal weight (g)	placental weight (g)	Fetal-to-placental weight ratio (g/g)
Control	6	11.6 ± 0.42	3.69 ± 0.05	0.597 ± 0.002	6.24 ± 0.20
TP0.5	6	11.1 ± 0.58	3.42 ± 0.05*	0.534 ± 0.003*	6.40 ± 0.11

Means ± S.E.M. *P < 0.05 *versus *control group.

#### Placental uptake and transport of 3-O-methyl-D-[^3^H]glucose and [^14^C]methylamino isobutyric acid (MeAIB)

**Figure 3:**
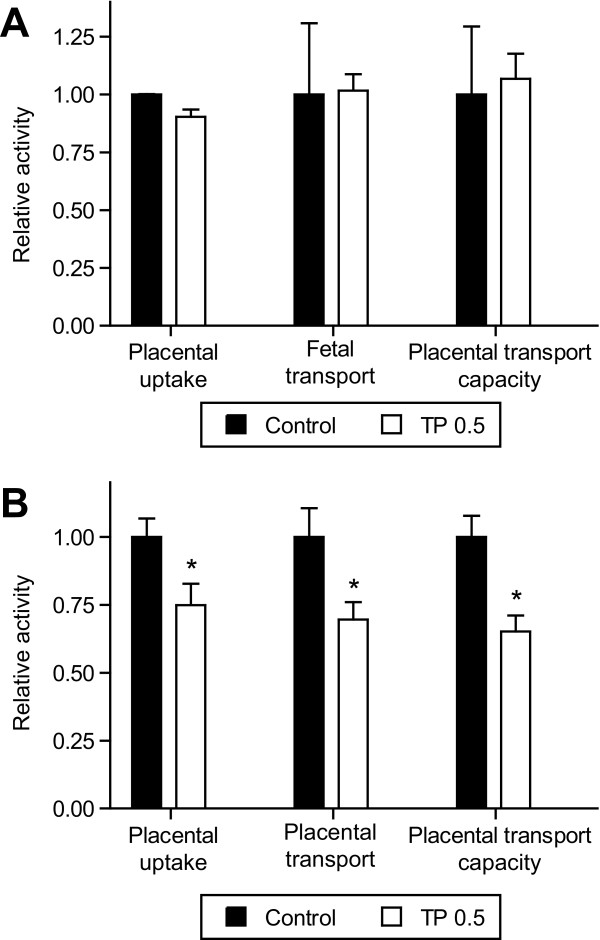
**Placental amino acid and glucose transport capacity in control and TP dams**. Placental Transport of (**A**) 3-*O*-methyl-D-[^3^H]glucose (MG) or (**B**) [^14^C]methylaminoisobutyric acid (MeAIB) to the fetus at GD 21. TP was administered prenatally (0.5 mg/kg; gestational days 15-19) and placental uptake (placental (dpm) per gram placenta), placental transport to the fetus (fetal dpm per gram fetus) and placental transport capacity (fetal dpm per gram placenta) of MG or MeAIB are expressed for the TP (n = 6) group in relation to control (n = 6) where control values are arbitrarily assigned a value of 1. Values are given as means ± S.E.M. *P < 0.05 vs controls.

The placental uptake (placental dpm per gram placenta) and transport to the fetus (fetal dpm per gram fetus) of 3-*O*-methyl-D-[^3^H]glucose were not altered by TP treatment. Similarly, the placental transport activity (fetal dpm per gram placenta) of 3-*O*-methyl-D-[^3^H]glucose was unchanged by TP treatment (Figure 3A). In contrast, placental uptake, placental transport, and placental transport capacity of MeAIB were significantly reduced (*P *< 0.05) by 20%, 26%, and 19%, respectively, in the TP group compared with controls (Figure 3B; n = 6 in each group).

#### Placental mRNA expression of Snat's

**Figure 4:**
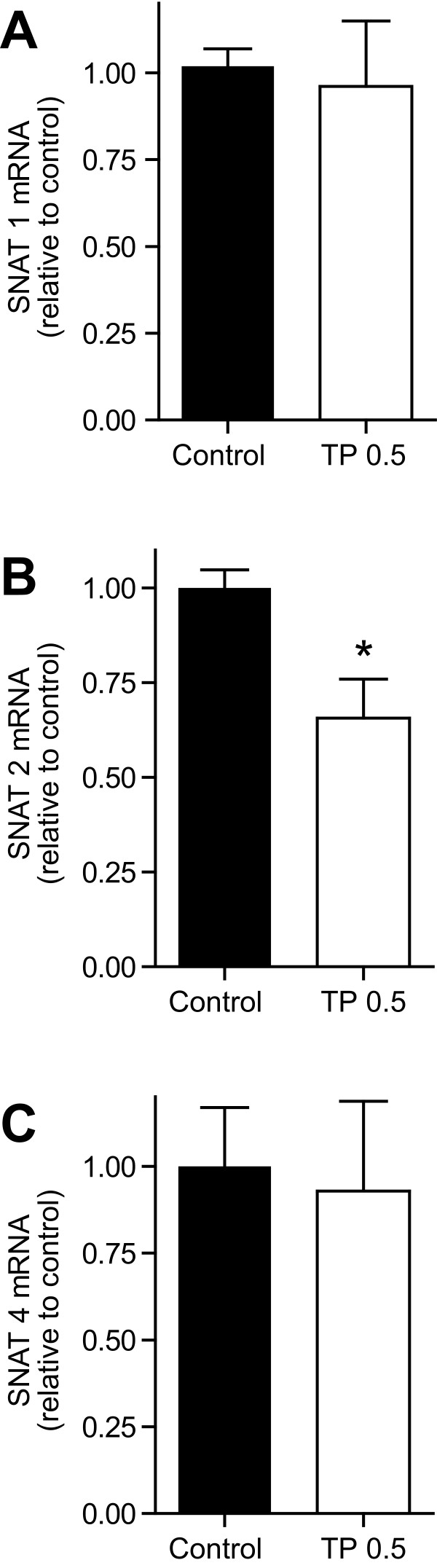
**Relative mRNA expression of system A amino acid transporters in placenta of control and TP dams**. TP was administered prenatally (0.5 mg/kg; gestational days 15-19) and (**A**) *slc38a1*/Snat1, (**B**) *slc38a2*/Snat2 and (**C**) *slc38a4*/Snat4 mRNA expression was assessed by real-time RT-PCR in the placenta obtained from control (n = 6) and TP (n = 6) dams on GD 21. The mean mRNA expression of the control group was assigned a value of 1 and the mean of the TP group was calculated relative to the control group. Values are given as mean ± SEM. *P < 0.05 *versus *control.

We next examined if the decrease in amino acid transport capacity is associated with a decreased expression of amino acid transporters. There are 3 known isoforms of system A present in the rat placenta, *slc38a1, 2*, and *4 *[43]. Treatment with TP significantly reduced the mRNA expression of *slc38a2 *(*P *< 0.05; n = 6 in each group) (Figure 4B) in GD 21 placenta compared to controls. However, mRNA expression of *slc38a1 *and *slc38a4 *was not affected at GD 21 in the TP group compared with the control group (Figure 4A and 4C).

#### Protein expression of Snat's

**Figure 5:**
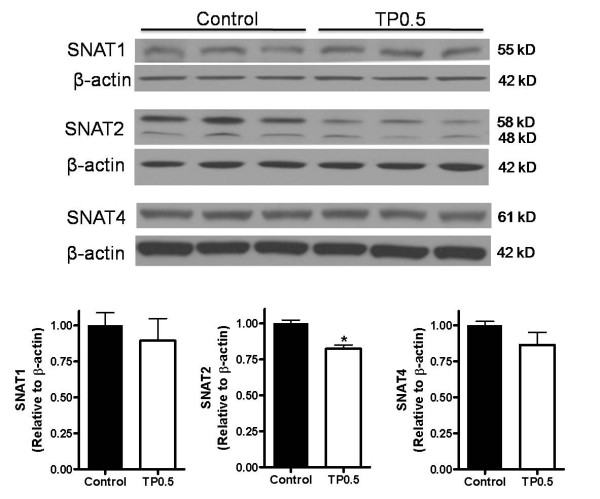
**Relative protein expression of system A amino acid transporters in placenta of control and TP dams**. TP was administered prenatally (0.5 mg/kg; gestational days 15-19) and protein expression, as measured by Western blot analysis in homogenates from rat placenta at GD 21 (n = 6, each group). Top panels show representative blots of the respective Snats and β-actin, and bottom panel is the summary of densitometric results. The mean density of the control group was assigned a value of 1, and the mean density of the TP group was calculated relative to the control group. Values are given as means ± SEM *P < 0.05 vs control.

We examined if the decreased mRNA levels of *slc38a2 *translated to reduced protein levels. Protein expression of Snat2 in placental homogenates was reduced at GD 21 by 18% (*P *< 0.05) in the TP group (n = 6) compared to control group (n = 6) (Figure 5). Snat2 antibody detected two distinct bands at approximately 58 and 48 kDa in Western blot. Antibody specificity was determined in preadsorption experiments, and both these bands were markedly attenuated (data not shown). For quantification, densitometry values of both these bands were analysed together. Protein expression of Snat1 and Smat4 in placental homogenates was similar at GD 21 in the TP and controls placental homogenates (Figure 5).

### Experiment 3: Birth weight and AGD

**Figure 6:**
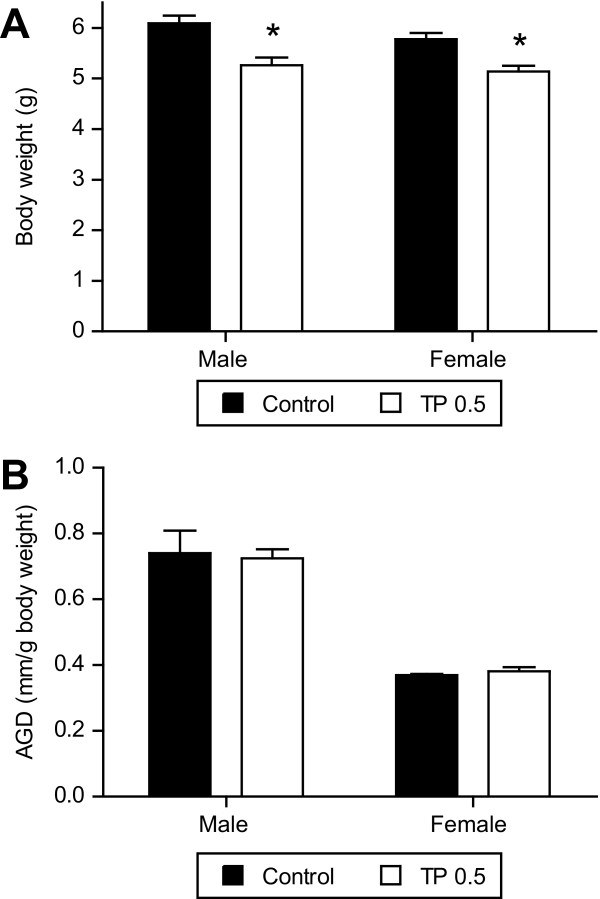
**Birth weight of pups and their anogenital distances in control and TP groups**. TP was administered prenatally (0.5 mg/kg; gestational days 15-19) and (**A**) birth weight (n = 6 litters, each group) and (**B**) anogenital distances were measured in both male and female pups at term delivery (n = 6 litters, each group). Data expressed as means ± SEM. *P < 0.05 vs controls.

Treatment of pregnant rats with TP caused a significant reduction (*P *< 0.05; n = 8, each litter) in birth weight of male and female pups by 14% and 11%, respectively, compared with their respective gender in the vehicle-treated group (Figure 6A). The AGD was larger in the males and shorter in the females in control and TP pups. Treatment by TP did not affect the mean AGD in pups of either sex, when compared to their respective gender in controls (Figure 6B).

## Discussion

The present study demonstrated that a 2-fold increase in maternal testosterone levels markedly reduced the birth weight of both males and females pups compared with controls. These findings indicate that elevated maternal plasma testosterone concentrations, similar to those observed in complicated human IUGR pregnancies [11-15], have placental and fetal growth inhibitory properties. Testosterone is a lipophilic hormone and is suggested to diffuse across tissues including placenta [24-26]; however increase in maternal plasma testosterone concentration, that caused fetal growth inhibition, was not associated with increase in fetal body testosterone concentration or alteration in AGD (functional indicator of androgenicity). This finding may indicate that testosterone administered to the dam is not delivered to the fetus, but is metabolized or blocked at the placenta [27,44]. Rat placenta does not have aromatase enzyme [45,46] to convert testosterone to estradiol but predominately expresses Δ^4^-reductase and 17β-hydroxysteroid dehydrogenase that metabolizes testosterone to weak androgens such as 5α-androstane-3α-17β-diol [47] and androsterone [27], respectively. Testosterone given to the pregnant rat may be metabolized by the dam, placenta and/or fetus to other androgens, of which androsterone is the most abundant in the fetus, followed by 3α-androstanediol and epiandrosterone [27]. Unlike rats, human and sheep placentae have aromatase enzyme that effectively metabolizes androgens to estrogens preventing fetal exposure [22,25]. Similar findings of a lack of increase in fetal testosterone levels, despite an increase in maternal testosterone levels, is reported in pregnant rats [27], guinea pigs [44], and sheep [22]. There are no reports on the fetal testosterone levels in human IUGR and preeclamptic pregnancies, however a 20-fold increase in maternal testosterone levels is not associated with changes in testosterone levels in cord venous blood [48]. It appears that the placenta is an effective barrier for testosterone under conditions of moderate increases in maternal testosterone concentrations. When the metabolizing capability of the enzymes is exceeded, a significant increase in fetal testosterone levels may occur, as observed in the dams exposed to higher maternal testosterone concentrations (TP 2.5 mg/kg group) in our study. This finding may mean that moderate increases in maternal testosterone concentrations, as observed in the TP 0.5 mg/kg group, and which occurs in most complicated human pregnancies, do not cause fetal growth restriction by their direct action on the fetus. In addition, testosterone is an anabolic hormone, and one would anticipate an increased testosterone concentration in the fetal environment to facilitate growth rather than having a negative effect on fetal growth. For example, testosterone exposure to newborn pups by injection has shown to accelerate growth rate [49]. These observations suggest that maternal testosterone does not cross the placenta to exert direct effect on fetal growth but rather may alter critical functions that support fetal growth.

The circulating levels of glucose, triglycerides, cholesterol, insulin, leptin, IGF-1 and thyroid hormones are unaltered in testosterone-exposed pregnant animals [22,23,30] suggesting that the observed effects of testosterone on fetal growth in this model may not be secondary to metabolic changes. Theoretically, increased maternal testosterone levels could reduce fetal growth and birth weight through impaired placental function. This finding is substantiated by diminished uteroplacental perfusion and low fetal birth weight by women who have increased testosterone levels due to impaired placental aromatization [50,51]. In mammals, the major determinant of intrauterine growth is the placental supply of nutrients to the fetus [52]. The capacity of the placenta to deliver nutrients to the fetus depends on a range of factors, including its size, morphology, blood flow, transporter abundance, and its rate of production of nutrients. The testosterone-exposed dams in this study have smaller placentas when compared with those of control dams. Similar reports of testosterone-induced reduction in placental weights are reported in rats [27]. The reason for smaller placenta in TP-exposed dams is not known but may involve increased apoptosis or decreased proliferation as reported in endothelial cells [53]. It is possible that high maternal testosterone leads to advanced placental differentiation contributing for alteration in placental weight/morphology [22].

One of the important functions of the placenta is to promote nutrient transport to the fetus. Glucose and amino acids are essential for the developing fetus and, as such, must be transported from the maternal circulation through their transporters. It is well established that the activity and/or expression of placental nutrient and ion transporters are modified in human pregnancies complicated by altered fetal growth [54]. The capacity of the placenta to transport glucose is not altered in human IUGR [55] but is increased in pregnancies complicated by type-1 diabetes [56], which is associated with accelerated fetal growth. In contrast, a number of placental transport systems for essential amino acids, such as transporters for lysine, leucine and taurine [57], are down-regulated in IUGR, whereas placental leucine transport activity is increased in accelerated fetal growth [58]. In the current study, glucose transport in vivo was unaffected in this model, findings that are in line with observations in human pregnancies complicated with fetal growth inhibition [59] and models of placental insufficiency in the rodent [60]. One placental amino acid transport system that has attracted particular interest in association with altered fetal growth is System A. In the current study, placental System A transport activity in vivo was significantly reduced in testosterone-exposed dams. Decreases in the mRNA and protein expression of *slc38a2*/Snat 2 (but not of *slc32a1*/Snat1 and *slc38a4*/Snat4) in placental homogenates at GD 21 suggest that the reduced availability of transporters may contribute to the observed transport changes. These findings are in line with observations that Snat2 appears to be a highly regulated Snat isoform, both in the placenta [61-63] and in other tissues and cells, such as the mammary gland [63] and 3T3-L1 adipocytes [64]. The cellular mechanisms by which testosterone down regulates placental *Slc38a2/*Snat2 remain to be fully established. Decrease in non-essential amino acids levels was found to be associated with increased expression of *Slc38a2/*Snat2 and increased transport of MeAIB [65]. If the reverse is true, that high amino acid concentrations down-regulate system A activity, it cannot be excluded that the moderate increase in essential amino acids (lysine, histidine, leucine, isoleucine, and arginine), may contribute to the down-regulation of *Slc38a2*/Snat2 expression and reduced MeAIB transport. Furthermore, testosterone regulates mTOR activity in prostate cancer cells [66]. Since mTOR signaling pathway regulates amino acid transport in the placenta and that the activity of the placental mTOR pathway is reduced in IUGR [67], it would be interesting to examine if testosterone affect mTOR signaling. It is also possible that testosterone may indirectly regulate the expression and function of transporters through other signaling mediators, such as glucocorticoids, insulin, growth hormone, and leptin [68]. Although we and others have shown that estradiol levels were unaltered in pregnant rats [17] and sheep [22] exposed to testosterone propionate, absence of measures of conjugated estradiol or estradiol metabolites cannot rule out the possible contribution of estradiol or their metabolites to fetal growth restriction. An increase in conjugated E_2 _in the maternal circulation, supportive of increased aromatization, has been reported in prenatal testosterone-treated monkeys [69].

An additional decrease in the transport of amino acids might occur because of a decreased availability of substrate to the transporters as a result of a decreased amino acid pool in the maternal circulation. Our studies show that maternal plasma amino acid concentrations are relatively maintained following testosterone exposure, with the exception of decreases in glutamine, glycine, tyrosine, serine, proline, and hydroxyproline and increases in asparagine, isoleucine, lysine, histidine, leucine and arginine. Studies have shown that testosterone promotes the increased utilization of hydroxyproline leading to greater collagen deposition in smooth muscle cells [70] which could have led to such dramatic reductions (-80%) in their plasma levels in TP exposed dams. We cannot conclusively say that changes in the supply of these amino acids are responsible for fetal growth restriction in TP- exposed dams, since a deficiency of one amino acid, in general, could be compensated for by others unless it is rate-limiting. On the other hand, studies have also demonstrated that suboptimal levels of even single amino acids, such as taurine, can impair normal fetal development [71]. These observations suggest that the down regulation of placental amino acid transporters, such as Snat2 and possibly the supply of amino acids, especially glycine, serine and glutamine which are substrates for system A transporter, may contribute for the decreased amino acid supply to the fetus and causes fetal growth restriction. It is surprising that, despite increases in five essential amino acids and no change in the majority of amino acids, that only reduction in four non-essential amino acids (transported by Snat2) is associated with growth restriction. It is possible that other essential amino acids transport mechanisms may also play a role in fetal/neonatal growth restriction. With a substantial reduction of the placental transport of neutral amino acids at GD 21, the rapid fetal growth (approximately 1 g day^-1^) at this stage of gestation is difficult to sustain, which could understandably lead to fetal growth restriction and delivery of low-birth-weight pups at term. The consistent finding of a relationship between placental System A and fetal growth in human pregnancy [72,73] and the IUGR observed as a result of inhibiting placental System A activity [74] support the important role of placental system A activity in determining fetal growth.

## Conclusions

In conclusion, the present study shows that an increase in maternal testosterone in concentrations commonly observed during pathological pregnancies caused a marked reduction in maternal weight gain through the dosing period until delivery and resulted in significantly smaller than normal fetuses. A 2-fold increase in maternal testosterone causes a significant decrease in fetal size but does not elevate fetal testosterone. Thus, the fetus appears to be protected from excess maternal androgen perhaps due to effective enzymatic inactivation by the placenta. Moreover, these studies demonstrate that the specific downregulation of amino acid transporters within the placenta in response to TP treatment is associated with a decrease in fetal growth. The decrease in growth may be the result of a decrease of available nutrients to the fetus.

## Competing interests

The authors declare that they have no competing interests.

## Authors' contributions

KS conceived and designed the study, performed the surgeries and hormone measurements, analyzed the data and wrote the manuscript, RE, HG and VC performed Western blot analysis and real-time PCR and organized the collection of tissues, GDVH and CY participated in experimental design, analysis and discussion of the results, and review of the manuscript. All authors read and approved the final manuscript.

## References

[B1] Wolf CJ Hotchkiss A Ostby JS LeBlanc GA Gray J Effects of prenatal testosterone propionate on the sexual development of male and female rats: A dose-response study Toxicol Sci 2002 65 71 86 10.1093/toxsci/65.1.71 11752687

[B2] Bruns CM Baum ST Colman RJ Eisner JR Kemnitz JW Weindruch R Abbott DH Insulin resistance and impaired insulin secretion in prenatally androgenized male rhesus monkeys J Clin Endocrinol Metab 2004 89 6218 6223 10.1210/jc.2004-0918 15579780

[B3] Manikkam M Crespi EJ Doop DD Herkimer C Lee JS Yu S Brown MB Foster DL Padmanabhan V Fetal Programming: Prenatal Testosterone Excess Leads to Fetal Growth Retardation and Postnatal Catch-Up Growth in Sheep Endocrinology 2004 145 790 798 14576190 10.1210/en.2003-0478

[B4] Crespi EJ Steckler TL MohanKumar PS Padmanabhan V Prenatal exposure to excess testosterone modifies the developmental trajectory of the insulin-like growth factor system in female sheep J Physiol 2006 572 119 130 16484301 10.1113/jphysiol.2005.103929 PMC1779643

[B5] Carlsen SM Jacobsen G Romundstad P Maternal testosterone levels during pregnancy are associated with offspring size at birth Eur J Endocrinol 2006 155 365 370 10.1530/eje.1.02200 16868152

[B6] Homburg R Pregnancy complications in PCOS Best Practice and Research: Clin Endocrinol Metab 2006 20 281 292 10.1016/j.beem.2006.03.009 16772158

[B7] Xita N Tsatsoulis A Review: Fetal programming of polycystic ovary syndrome by androgen excess: Evidence from experimental, clinical, and genetic association studies J Clin Endocrinol Metab 2006 91 1660 1666 10.1210/jc.2005-2757 16522691

[B8] Acromite MT Mantzoros CS Leach RE Hurwitz J Dorey LG Androgens in preeclampsia Am J Obstet Gynecol 1999 180 60 63 10.1016/S0002-9378(99)70150-X 9914579

[B9] Salamalekis E Bakas P Vitoratos N Eleptheriadis M Creatsas G Androgen levels in the third trimester of pregnancy in patients with preeclampsia Eur J Obstet Gynecol Reprod Biol 2006 126 16 19 10.1016/j.ejogrb.2005.07.007 16139944

[B10] Ghorashi V Sheikhvatan M The relationship between serum concentration of free testosterone and pre-eclampsia Endokrynol Pol 2008 59 390 392 18979448

[B11] Bastek JA Pare E Wang E Elovitz MA Srinivas SK Limitations of ultrasound in diagnosing intrauterine growth restriction in severe preeclampsia J Matern Fetal Neonatal Med 2009 22 1039 1044 10.3109/14767050903029584 19900041

[B12] Rahimi R Nikfar S Rezaie A Abdollahi M A meta-analysis on the efficacy and safety of combined vitamin C and E supplementation in preeclamptic women Hypertens Pregnancy 2009 28 417 434 10.3109/10641950802629667 19843004

[B13] Francavilla F Zugaro A Pandolfi C Lattanzio F Necozione S Anselmi M Francavilla S Low birth weight either small (SGA) or appropriate for gestational age (AGA) and later development of insulin resistance and clinical features of PCOS Hum Reprod 2007 22 s188 189

[B14] Nawaz FH Khalid R Naru T Rizvi J Does continuous use of metformin throughout pregnancy improve pregnancy outcomes in women with polycystic ovarian syndrome? J Obstet Gynaecol Res 2008 34 832 837 10.1111/j.1447-0756.2008.00856.x 18834342

[B15] Sir-Petermann T Hitchsfeld C Maliqueo M Codner E Echiburu B Gazitua R Recabarren S Cassorla F Birth weight in offspring of mothers with polycystic ovarian syndrome Hum Reprod 2005 20 2122 2126 10.1093/humrep/dei009 15802312

[B16] Bremner WJ Cumming IA Inhibition of fetal growth and survival by testosterone administration to pregnant sheep Metabolism: Clinical and Experimental 1978 27 253 255 10.1016/0026-0495(78)90104-x 628349

[B17] Sathishkumar K Elkins R Yallampalli U Balakrishnan M Yallampalli C Fetal programming of adult hypertension in female rat offspring exposed to androgens in utero Early Hum Dev 2011 87 407 414 10.1016/j.earlhumdev.2011.03.001 21450421 PMC3093104

[B18] Geelhoed JJ Fraser A Tilling K Benfield L Davey SG Sattar N Nelson SM Lawlor DA Preeclampsia and gestational hypertension are associated with childhood blood pressure independently of family adiposity measures: the Avon Longitudinal Study of Parents and Children Circulation 2010 122 1192 1199 10.1161/CIRCULATIONAHA.110.936674 20823385 PMC5321267

[B19] Jayet PY Rimoldi SF Stuber T Salmon CS Hutter D Rexhaj E Thalmann S Schwab M Turini P Sartori-Cucchia C Nicod P Villena M Allemann Y Scherrer U Sartori C Pulmonary and systemic vascular dysfunction in young offspring of mothers with preeclampsia Circulation 2010 122 488 494 10.1161/CIRCULATIONAHA.110.941203 20644018

[B20] Luft FC Pre-eclampsia and the maternal cardiovascular risk Nephrol Dial Transplant 2003 18 860 861 10.1093/ndt/gfg062 12686652

[B21] Laivuori H Kaaja R Rutanen EM Viinikka L Ylikorkala O Evidence of high circulating testosterone in women with prior preeclampsia J Clin Endocrinol Metab 1998 83 344 347 10.1210/jc.83.2.344 9467538

[B22] Veiga-Lopez A Steckler TL Abbott DH Welch KB MohanKumar PS Phillips DJ Refsal K Padmanabhan V Developmental programming: impact of excess prenatal testosterone on intrauterine fetal endocrine milieu and growth in sheep Biol Reprod 2011 84 87 96 10.1095/biolreprod.110.086686 20739662 PMC3012564

[B23] Sathishkumar K Gao HJ Yallampalli C Cardiovascular but not the metabolic functions are altered in pregnant rats with elevated testosterone levels [Abstract] Reprod Sci 2010 17 s789 10.1177/1933719110380396

[B24] Meulenberg PM Hofman JA Maternal testosterone and fetal sex J Steroid Biochem Mol Biol 1991 39 51 54 10.1016/0960-0760(91)90012-T 2069866

[B25] Dell'Acqua S Mancuso S Eriksson G Diczfalusy E Metabolism of retrotestosterone and testosterone by midterm human placentas perfused in situ BBA - Gen Sub 1966 130 241 248 10.1016/0304-4165(66)90028-6

[B26] Wang YC Su HY Liu JY Chang FW Chen CH Maternal and female fetal virilization caused by pregnancy luteomas Fertil Steril 2005 84 509.e15 e17 10.1016/j.fertnstert.2005.02.029 16086574

[B27] Slob AK den HR Woutersen PJ van der Werff ten Bosch JJ Prenatal testosterone propionate and postnatal ovarian activity in the rat Acta Endocrinol (Copenh) 1983 103 420 427 10.1530/acta.0.1030420 6880571

[B28] Desoye G Hauguel-De Mouzon S The human placenta in gestational diabetes mellitus: The insulin and cytokine network Diabetes Care 2007 30 s120 126 10.2337/dc07-s203 17596459

[B29] Weiss B Faus H Haendler B Phylogenetic conservation of the androgen receptor AR45 variant form in placental mammals Gene 2007 399 105 111 10.1016/j.gene.2007.04.037 17574777

[B30] Sathishkumar K Elkins R Yallampalli U Yallampalli C Elevated Androgen Levels During Pregnancy Impair Fetal Growth Due to Placental Insufficiency and Programs for Adult Hypertension in Rats Biol Reprod 2009 83 s103 104

[B31] Serin IS Kula M Basbug M Unluhizarci K Gucer S Tayyar M Androgen levels of preeclamptic patients in the third trimester of pregnancy and six weeks after delivery Acta Obstet Gynecol Scand 2001 80 1009 1013 10.1034/j.1600-0412.2001.801107.x 11703197

[B32] Salamalekis E Bakas P Vitoratos N Eleptheriadis M Creatsas G Androgen levels in the third trimester of pregnancy in patients with preeclampsia Eur J Obstet Gynecol Reprod Biol 2006 126 16 19 10.1016/j.ejogrb.2005.07.007 16139944

[B33] Rhees RW Kirk BA Sephton S Lephart ED Effects of prenatal testosterone on sexual behavior, reproductive morphology and LH secretion in the female rat Dev Neurosci 1997 19 430 437 10.1159/000111240 9323463

[B34] Sommerville EM Tarttelin MF Plasma testosterone levels in adult and neonatal female rats bearing testosterone propionate-filled silicone elastomer capsules for varying periods of time J Endocrinol 1983 98 365 371 10.1677/joe.0.0980365 6619712

[B35] McIntyre BS Barlow NJ Foster PM Androgen-mediated development in male rat offspring exposed to flutamide in utero: permanence and correlation of early postnatal changes in anogenital distance and nipple retention with malformations in androgen-dependent tissues Toxicol Sci 2001 62 236 249 10.1093/toxsci/62.2.236 11452136

[B36] Kuddus RH Lee YH Valdivia LA A semiquantitative PCR technique for detecting chimerism in hamster-to-rat bone marrow xenotransplantation J Immunol Methods 2004 285 245 251 10.1016/S0022-1759(03)00250-3 14980438

[B37] Parks LG Ostby JS Lambright CR Abbott BD Klinefelter GR Barlow NJ Gray LE Jr The plasticizer diethylhexyl phthalate induces malformations by decreasing fetal testosterone synthesis during sexual differentiation in the male rat Toxicol Sci 2000 58 339 349 10.1093/toxsci/58.2.339 11099646

[B38] Coan PM Angiolini E Sandovici I Burton GJ Constancia M Fowden AL Adaptations in placental nutrient transfer capacity to meet fetal growth demands depend on placental size in mice J Physiol 2008 586 4567 4576 10.1113/jphysiol.2008.156133 18653658 PMC2614013

[B39] Ericsson A Saljo K Sjostrand E Jansson N Prasad PD Powell TL Jansson T Brief hyperglycaemia in the early pregnant rat increases fetal weight at term by stimulating placental growth and affecting placental nutrient transport J Physiol 2007 581 1323 1332 10.1113/jphysiol.2007.131185 17430988 PMC2170823

[B40] Jansson N Pettersson J Haafiz A Ericsson A Palmberg I Tranberg M Ganapathy V Powell TL Jansson T Down-regulation of placental transport of amino acids precedes the development of intrauterine growth restriction in rats fed a low protein diet J Physiol 2006 576 935 946 16916910 10.1113/jphysiol.2006.116509 PMC1892642

[B41] Guzman C Cabrera R Cardenas M Larrea F Nathanielsz PW Zambrano E Protein restriction during fetal and neonatal development in the rat alters reproductive function and accelerates reproductive ageing in female progeny J Physiol 2006 572 97 108 16497715 10.1113/jphysiol.2005.103903 PMC1779641

[B42] Baum MJ Woutersen PJ Slob AK Sex difference in whole-body androgen content in rats on fetal days 18 and 19 without evidence that androgen passes from males to females Biol Reprod 1991 44 747 751 10.1095/biolreprod44.5.747 1868134

[B43] Mackenzie B Erickson JD Sodium-coupled neutral amino acid (System N/A) transporters of the SLC38 gene family Pflugers Archiv-Eur J Physiol 2004 447 784 795 10.1007/s00424-003-1117-9 12845534

[B44] Vreeburg JT Woutersen PJ Ooms MP van der Werff ten Bosch JJ Androgens in the fetal guinea-pig after maternal infusion of radioactive testosterone J Endocrinol 1981 88 9 16 10.1677/joe.0.0880009 7462896

[B45] Akinola LA Poutanen M Peltoketo H Vihko R Vihko P Characterization of rat 17 beta-hydroxysteroid dehydrogenase type 1 gene and mRNA transcripts Gene 1998 208 229 238 10.1016/S0378-1119(97)00669-0 9524272

[B46] Lephart ED Herbst MA McPhaul MJ Characterization of aromatase cytochrome P-450 mRNA in rat perinatal brain, ovary and a Leydig tumor cell line: evidence for the existence of brain specific aromatase transcripts Endocrine 1995 3 25 31 10.1007/BF02917445 21153233

[B47] Sybulski S Testosterone metabolism by rat placenta Steroids 1969 14 427 440 10.1016/S0039-128X(69)80005-X 4390509

[B48] Simmer HH Frankland MV Greipel M Neutral C19-steroids and steroid sulfates in human pregnancy: VII. Plasma testosterone in maternal peripheral blood and in cord venous blood after administration of testosterone enanthate to the mother Steroids 1972 19 229 242 10.1016/0039-128X(72)90007-4 5013449

[B49] Slob AK van der Werff ten Bosch JJ Sex differences in body growth in the rat Physiol Behav 1975 14 353 361 10.1016/0031-9384(75)90044-X 1169787

[B50] Tanguy G Thoumsin HJ Zorn JR Cedard L DHEA-S-loading test in cases of intrauterine growth retardation: relationship between the pattern of the maternal plasma metabolites and the fetoplacental dysfunction Gynecol Obstet Invest 1981 12 305 316 10.1159/000299660 6457777

[B51] Thoumsin HJ Alsat E Cedard L In vitro aromatization of androgens into estrogens in placental insufficiency Gynecol Obstet Invest 1982 13 37 43 10.1159/000299482 6459977

[B52] Harding JE Johnston BM Nutrition and fetal growth Reprod Fertil Dev 1995 7 539 547 10.1071/RD9950539 8606966

[B53] Ling S Dai A Williams MR Myles K Dilley RJ Komesaroff PA Sudhir K Testosterone (T) enhances apoptosis-related damage in human vascular endothelial cells Endocrinology 2002 143 1119 1125 10.1210/en.143.3.1119 11861539

[B54] Jansson T Powell TL Placental nutrient transfer and fetal growth Nutrition 2000 16 500 502 10.1016/S0899-9007(00)00323-3 10906535

[B55] Jansson T Wennergren M Illsley NP Glucose transporter protein expression in human placenta throughout gestation and in intrauterine growth retardation J Clin Endocrinol Metab 1993 77 1554 1562 10.1210/jc.77.6.1554 8263141

[B56] Jansson T Ekstrand Y Wennergren M Powell TL Placental glucose transport in gestational diabetes mellitus Am J Obstet Gynecol 2001 184 111 116 10.1067/mob.2001.108075 11174489

[B57] Jansson T Scholtbach V Powell TL Placental transport of leucine and lysine is reduced in intrauterine growth restriction Pediatr Res 1998 44 532 537 10.1203/00006450-199810000-00011 9773842

[B58] Jansson T Ekstrand Y Bjorn C Wennergren M Powell TL Alterations in the activity of placental amino acid transporters in pregnancies complicated by diabetes Diabetes 2002 51 2214 2219 10.2337/diabetes.51.7.2214 12086952

[B59] Jansson T Ylven K Wennergren M Powell TL Glucose transport and system A activity in syncytiotrophoblast microvillous and basal plasma membranes in intrauterine growth restriction Placenta 2002 23 392 399 10.1053/plac.2002.0826 12061855

[B60] Jansson T Persson E Placental-Transfer of Glucose and Amino-Acids in Intrauterine Growth-Retardation - Studies with Substrate-Analogs in the Awake Guinea-Pig Pediatr Res 1990 28 203 208 2235115 10.1203/00006450-199009000-00007

[B61] Jones HN Woollett LA Barbour N Prasad PD Powell TL Jansson T High-fat diet before and during pregnancy causes marked up-regulation of placental nutrient transport and fetal overgrowth in C57/BL6 mice FASEB J 2009 23 271 278 10.1096/fj.08-116889 18827021 PMC2626621

[B62] Nelson DM Smith SD Furesz TC Sadovsky Y Ganapathy V Parvin CA Smith CH Hypoxia reduces expression and function of system A amino acid transporters in cultured term human trophoblasts Am J Physiol Cell Physiol 2003 284 C310 C315 12388062 10.1152/ajpcell.00253.2002

[B63] Lopez A Torres N Ortiz V Aleman G Hernandez-Pando R Tovar AR Characterization and regulation of the gene expression of amino acid transport system A (SNAT2) in rat mammary gland Am J Physiol Endocrinol Metab 2006 291 E1059 E1066 10.1152/ajpendo.00062.2006 16787963

[B64] Hatanaka T Hatanaka Y Tsuchida J Ganapathy V Setou M Amino acid transporter ATA2 is stored at the trans-Golgi network and released by insulin stimulus in adipocytes J Biol Chem 2006 281 39273 39284 10.1074/jbc.M604534200 17050538

[B65] Jones HN Ashworth CJ Page KR McArdle HJ Expression and adaptive regulation of amino acid transport system A in a placental cell line under amino acid restriction Reproduction 2006 131 951 960 10.1530/rep.1.00808 16672359

[B66] Wu Y Chhipa RR Cheng J Zhang H Mohler JL Ip C Androgen receptor-mTOR crosstalk is regulated by testosterone availability: implication for prostate cancer cell survival Anticancer Res 2010 30 3895 3901 21036700 PMC4355915

[B67] Roos S Jansson N Palmberg I Saljo K Powell TL Jansson T Mammalian target of rapamycin in the human placenta regulates leucine transport and is down-regulated in restricted fetal growth J Physiol 2007 582 449 459 10.1113/jphysiol.2007.129676 17463046 PMC2075295

[B68] Fowden AL Ward JW Wooding FP Forhead AJ Constancia M Programming placental nutrient transport capacity J Physiol 2006 572 5 15 16439433 10.1113/jphysiol.2005.104141 PMC1779642

[B69] Abbott DH Barnett DK Levine JE Padmanabhan V Dumesic DA Jacoris S Tarantal AF Endocrine Antecedents of Polycystic Ovary Syndrome in Fetal and Infant Prenatally Androgenized Female Rhesus Monkeys Biol Reprod 2008 79 154 163 10.1095/biolreprod.108.067702 18385445 PMC2531213

[B70] Leitman DC Benson SC Johnson LK Glucocorticoids stimulate collagen and noncollagen protein synthesis in cultured vascular smooth muscle cells J Cell Biol 1984 98 541 549 10.1083/jcb.98.2.541 6693495 PMC2113086

[B71] Sturman JA Taurine in development J Nutr 1988 118 1169 1176 3054019 10.1093/jn/118.10.1169

[B72] Dicke JM Henderson GI Placental Amino-Acid Uptake in Normal and Complicated Pregnancies Am J Med Sci 1988 295 223 227 10.1097/00000441-198803000-00012 3354595

[B73] Mahendran D Donnai P Glazier JD Dsouza SW Boyd RDH Sibley CP Amino-Acid (System-A) Transporter Activity in Microvillous Membrane-Vesicles from the Placentas of Appropriate and Small-For-Gestational-Age Babies Pediatr Res 1993 34 661 665 8284106 10.1203/00006450-199311000-00019

[B74] Cramer S Beveridge M Kilberg M Novak D Physiological importance of system A-mediated amino acid transport to rat fetal development Am J Physiol Cell Physiol 2002 282 C153 C160 11742808 10.1152/ajpcell.2002.282.1.C153

